# Maternal iron-deficiency is associated with premature birth and higher birth weight despite routine antenatal iron supplementation in an urban South African setting: The NuPED prospective study

**DOI:** 10.1371/journal.pone.0221299

**Published:** 2019-09-03

**Authors:** Elizabeth A. Symington, Jeannine Baumgartner, Linda Malan, Amy J. Wise, Cristian Ricci, Lizelle Zandberg, Cornelius M. Smuts

**Affiliations:** 1 Centre of Excellence for Nutrition, North-West University, Potchefstroom, South Africa; 2 Department of Life and Consumer Sciences, University of South Africa, Johannesburg, South Africa; 3 Human Nutrition Laboratory, Institute of Food, Nutrition and Health, ETH Zürich, Switzerland; 4 Department of Obstetrics and Gynaecology, University of the Witwatersrand, Johannesburg, South Africa; 5 Empilweni Services and Research Unit, University of the Witwatersrand, Johannesburg, South Africa; Metrohealth Medical Center, UNITED STATES

## Abstract

**Background:**

Recent studies are suggesting a U-shaped relationship between antenatal iron exposure and birth outcomes. Little is known about the iron status and associated birth outcomes of pregnant women in South Africa. Our aim was to assess iron status at early, mid- and late pregnancy, and to determine associations with gestational age and birth weight in women in Johannesburg, South Africa.

**Methods:**

In this prospective study of 250 pregnant women, we measured haemoglobin, biomarkers of iron status and inflammation at <18, 22 and 36 weeks of gestation, plus birth weight and gestational age at delivery. Associations of anaemia and iron status with birth outcomes were determined using regression models adjusted for confounders.

**Results:**

At enrolment, the prevalence of anaemia, iron depletion (ID) and iron deficiency erythropoiesis (IDE) was 29%, 15% and 15%, respectively, and increased significantly with pregnancy progression. Anaemia and ID at 22 weeks, as well as IDE at 36 weeks were associated with higher birth weight (β = 135.4; 95% CI: 4.8, 266.1 and β = 205.4; 95% CI: 45.6, 365.1 and β = 178.0; 95% CI: 47.3, 308.7, respectively). Women in the lowest ferritin quartile at 22 weeks gave birth to babies weighing 312 g (95% CI: 94.8, 528.8) more than those in the highest quartile. In contrast, IDE at 22 weeks was associated with a higher risk for premature birth (OR: 3.57, 95% CI: 1.24, 10.34) and women in lower haemoglobin quartiles at <18 weeks had a shorter gestation by 7 days (β = -6.9, 95% CI: -13.3, -0.6) compared to those in the highest quartile.

**Conclusion:**

Anaemia, ID and IDE prevalence increased during pregnancy despite routine iron supplementation. ID and anaemia at mid-pregnancy were associated with higher birth weight, while IDE was associated with premature birth. These results suggest that current antenatal screening and supplementation practices in South Africa need to be revisited.

## Introduction

Iron deficiency anaemia (IDA) is the result of prolonged iron depletion and affects a third of the world’s population [1]. Pregnancy is a period of increased physiological iron requirements, and a deficiency may have variable effects on pregnancy, maternal and child health outcomes depending on the severity and time point of occurrence [2]. Preconception IDA has been associated with reduced infant growth and increased risk of adverse pregnancy outcomes [3], while IDA in the third trimester has been associated with poorer mental development of the child [4]. More recently, U-shaped associations of maternal haemoglobin and serum ferritin concentrations with risk for low birth weight and preterm birth have received heightened attention [5,6].

Data on iron intake and status of pregnant women in South Africa are sparse. National data on anaemia and iron status are available for women of reproductive age only. The South Africa Demographic and Health Survey conducted in 2016 estimated that 33% of women of reproductive age (15–49 years) are anaemic [7]. In the 2012 South African National Health and Nutrition Examination Survey (SANHANES) conducted among younger women (15 to 35 years) the prevalence of anaemia, iron depletion (ID) and IDA was 23%, 16% and 10%, respectively [8]. From these national data and a systematic review [9], approximately 23% to 33% of South African women are expected to enter pregnancy anaemic, and about half of these cases may be attributed to iron deficiency.

The South African Government is addressing iron deficiency in several ways. Firstly, the Government introduced mandatory fortification of maize meal and wheat flour with eight micronutrients, including iron (35 mg electrolytic iron per kg), in 2003 [10]. Secondly, and more specifically to improve status in pregnant women, the *Guidelines for maternity care in South Africa* [11] recommend routine nutritional assessment as well as daily supplementation with 200 mg ferrous sulphate (±65 mg of elemental iron), 1000 mg calcium and 5 mg folic acid. Regimes in South Africa may differ per province and pregnant women in Johannesburg (current study setting) receive 170 mg ferrous sulphate (±55 mg elemental iron) with calcium and folic acid. Supplementation is supplied to all pregnant women irrespective of individual iron or anaemia status. However, the effectiveness of routine antenatal iron supplementation in preventing anaemia and iron deficiency during pregnancy has not been evaluated nationally. A cross-sectional study at a regional hospital in Durban, South Africa, reported an anaemia (haemoblogin <11 g/dL) prevalence of 43% in 2000 pregnant women between 34 and 36 weeks of gestation, despite receiving routine iron supplements [12].

Determining haemoglobin concentrations forms part of the routine nutritional assessment during antenatal care, while iron status is only further investigated if referred by a physician. Since haemoglobin is not a sensitive marker of iron status, the iron status of pregnant women in South Africa is not well described. Thus, the current study was motivated by the limited evidence on the effectiveness of routine iron supplementation in iron replete pregnant women [13] as well as the limited data available on pregnant women in South Africa. Therefore, the aim of our study was to assess iron status at early, mid- and late pregnancy, and to determine associations with both birth weight and gestational age in urban pregnant women in Johannesburg, South Africa. In an effort to explain the observed iron status, we additionally assessed iron intake during early pregnancy.

## Materials and methods

### Study design and participants

This study formed part of the *Nutrition during Pregnancy and Early Development* (NuPED) study, which is a prospective study conducted in South Africa’s largest city, Johannesburg. The NuPED study protocol has been published previously [14]. Briefly, generally healthy pregnant women were recruited from primary healthcare clinics in Johannesburg between March 2016 and November 2017. Women were eligible for inclusion if they were aged 18–39 years, <18 weeks of gestation with singleton pregnancies, proficient in local languages, born in South Africa or neighbouring countries, and if they have been residing in Johannesburg for at least 12 months. Women were excluded if they reported using illicit drugs, were smoking, or had been diagnosed with a non-communicable disease (namely diabetes, renal disease, high cholesterol, and hypertension), an infectious disease (namely tuberculosis and hepatitis), or a serious illness (namely cancer, lupus or psychosis). Due to South Africa’s high prevalence of HIV infection (36% of women aged 30–34 years [15]), women who were HIV positive were included in the study in order for it to be a better representation of the general population. The volunteering women who agreed to participate were followed-up at the antenatal clinic of an academic hospital until June 2018. Data were collected at early pregnancy (<18 weeks of gestation), mid-pregnancy (±22 weeks), late pregnancy (±36 weeks) and at birth.

### Outcome measurements

The primary outcome measures were birth weight and gestational age at birth. At birth, four trained study nurses obtained neonatal weight (to the nearest 10g) using calibrated digital infant scales within 12 hours of birth [16]. In case the study nurse could not obtain the birth weight herself, it was obtained from the medical record (measured using the same calibrated scales). Low birth weight (LBW) was defined as birth weight <2500 g [17]. Date and time of birth were recorded from maternal records. Women who delivered elsewhere were followed-up telephonically to obtain baby’s date of birth and sex of the baby. Gestational age at birth was calculated in days using gestational age determined at the first visit, which was before 18 weeks gestation (minimum -maximum range: 6–17 weeks), by means of foetal ultrasonography examination using international recommendations [18]. Preterm birth was defined as birth <37 + 0 weeks of gestation (259 days) [19].

### Exposure measurements

#### Dietary and supplemented iron intake

Maternal dietary intake data were obtained at the first visit (<18 weeks of gestation) by means of an interviewer administered quantified food frequency questionnaire (QFFQ) using standardised probing questions [20]. The QFFQ was validated for a previous South African study [21], and its reproducibility was proven in similar study populations [22,23]. Women were asked according to the ~140 food items listed in the QFFQ, cooking methods, the type/brand, frequency and the amount of all food and beverages consumed in the past four weeks. To assist in portion size quantification, standard measuring equipment, two- and three-dimensional food models and common size containers (e.g. cups, bowls and glasses) were used. Three registered dietitians/nutritionists converted reported intakes to grams per week per food item using the Condensed Food Composition Tables for South Africa [24] and the South African Medical Research Council (SAMRC) Food Quantities Manual [25]. Analyses were done by the SAMRC by linking dietary intake data to the most recent food composition database to determine total daily dietary iron intake levels. The database includes the iron content values of fortified foods as per the food fortification programme. The Estimated Average Requirement (EAR) cut-point method was used to determine the proportion of subjects with intake below the EAR, indicative of inadequate intake of iron in this population.

Supplement use was determined from participants’ daily recorded supplement use (yes/no) on a supplied calendar from enrolment until birth. In addition, at each visit, the women were asked the type/brand, frequency and the amount of all dietary supplements used in the past week, taking into consideration supplementation supplied as part of routine care as well as store bought supplements. From these data, average daily iron intake from routine supplements and total supplements during pregnancy were calculated. Percentage compliance with routine supplementation was calculated as total reported routine supplemented iron intake divided by total routine iron supplied X 100. Routine iron supplementation included 55 mg elemental iron per day provided as 170 mg dried ferrous sulphate.

#### Haematological biomarkers

Maternal venous blood was drawn into labelled EDTA-coated and serum evacuated tubes at each visit during pregnancy. Haemoglobin concentrations were determined in whole blood (20μL) using calibrated HemoCue haemoglobin meters (Hb 201+, Ängelholm, Sweden). Haemoglobin values were adjusted for altitude as Johannesburg is located at 1753 meters above sea level [1]. Anaemia was defined as haemoglobin <11 g/dL at <18 weeks of gestation and haemoglobin <10.5 g/dL for mid- and late pregnancy based on cut-offs per trimester [26,27]. In addition, for the purpose of comparability, the prevalence of anaemia is reported according to the WHO [1] haemoglobin cut-off (<11 g/dL) throughout pregnancy. In cases where severe anaemia (haemoglobin <7 g/dL) was detected, the women were referred to the medical doctor on site and treated according to maternity care guidelines [11]. The treatment entailed higher doses of oral iron supplementation and these cases were therefore retained in analyses. In this study no women received parenteral iron therapy or blood transfusion.

Serum was separated within 1h after blood draw and stored at -20°C for a maximum of 14 days until transportation for storage at -80°C until analysis. Ferritin and soluble transferrin receptor (sTfR) concentrations were determined using the Q-Plex Human Micronutrient Array (7-plex, Quansys Bioscience, Logan, UT, USA) [28]. This fully quantitative chemiluminescent multiplex assay also includes the acute phase proteins C-reactive protein (CRP) and α_1_-acid glycoprotein (AGP). Ferritin concentrations were adjusted for inflammation using the correction factors recommended by Thurnham et al. [29]. Iron depletion (ID) was defined as adjusted ferritin <15 μg/L [30]. Iron deficient erythropoiesis (IDE) was defined as sTfR >8.3 mg/L [31]. Iron deficiency anaemia (IDA) was defined as ferritin <15 μg/L plus haemoglobin <11 g/dL [1].

### Covariates

Socio-economic and -demographic data, including maternal age and living standards measurements (reflective of socio-economic status) [32], were collected at the first visit early in pregnancy by means of an interviewer-administered questionnaire. Maternal anthropometric measurements (height and weight) were obtained using standardised methods from the International Society for the Advancement of Kinanthropometry [33] at each study visit. To determine body mass index (BMI) weight (kg) was divided by height (m) squared. An obstetrician conducted foetal ultrasonography examination to confirm gestational age and singleton pregnancy at the first visit [34,35]. A 2-hour 75 g oral glucose tolerance test was performed between 24 and 28 weeks of gestation using standard procedures [36]. Medical files were inspected to obtain data on maternal medical history, including parity, HIV status, mode of delivery, labour induction, as well as sex of the baby. During analyses, women were considered HIV positive irrespective of date of HIV contraction (prior to or during pregnancy).

### Statistical methods

Sample size calculation was done using the G*Power 3.1.9.2 statistical programme [37]. The calculation was based on multiple linear regression analysis (fixed model, single regression coefficient); a small effect size F^2^ of 0.05; probability of error (alpha) of 5%; a power of 80% and 10 predictors with birth weight as outcome. The result indicated a required sample size of 196 pregnant women. Considering an attrition rate of 25%, a minimum of 245 women were required. The sample size for this study was 250.

Data processing and statistical analysis of data were performed using SPSS version 25 (SPSS Inc, Chicago, IL, USA). Raw data were captured in Microsoft Access (Microsoft Corporation, Washington, USA) and 20% of all captured data were randomly checked for correctness. Dietary data were captured in Microsoft Excel (Microsoft Corporation, Washington, USA) and all electronic entries were double checked for the correct food code and a realistic amount captured.

Data were tested for outliers and normality by means of Q-Q plots, histograms and Shapiro-Wilk test. Normally distributed data are expressed as means ± SD; non-normally distributed data are expressed as medians (25th percentile - 75th percentile), except in the second results table which displays medians with minimum–maximum ranges. Descriptive statistics were conducted to describe iron intake at early pregnancy. To examine the longitudinal trajectory of the iron status parameters with pregnancy progression, median concentrations were determined at each visit.

Univariable analyses per outcome were performed using Mann-Whitney U-test for continuous variables and Chi-square test for categorical variables. To test for significance of change in haematological biomarkers (haemoglobin, ferritin, sTfR, CRP and AGP) over time we used the 2-tailed paired *t* test. For the significance of change in proportions for the conditions (anaemia, ID, IDE, IDA and inflammation) over time we used the McNemar test. Next we used logistic regression analyses to investigate the relationship between the exposure (haemoglobin, ferritin, sTfR) and outcome variables (low birth weight and preterm birth) as binary outcomes with odds ratios (OR) and 95% confidence intervals (CI). Multiple linear regression analyses were conducted for continuous outcome variables (birth weight in grams and gestational age at birth in days). The β coefficient was reported with 95% CIs. In both regression analyses, 3 models were applied and different sets of covariates for the two outcome variables. For birth weight, model 1 adjusted for maternal age, gestational age at birth and sex of the baby. Model 2 included the covariates of model 1 plus parity and socio-economic status. Model 3 included the covariates of models 1 and 2 plus HIV status, maternal BMI at enrolment and glucose tolerance. For gestational age at birth, model 1 adjusted for maternal age, baby sex and delivery intervention (induction or caesarean section). Model 2 adjusted additionally for parity and socio-economic status. Model 3 adjusted in addition to models 1 and 2 for HIV status, maternal BMI at enrolment and glucose tolerance. Lastly, univariate comparisons were done between quartiles of each iron biomarker adjusted for the same covariates as with the regression analyses. *P* values of <0.05 were considered significant.

### Ethical considerations

During recruitment, an informed consent form was supplied to potentially eligible women who were interested in being part of the study. Written informed consent was obtained at the first visit from all the women before data collection. This study was conducted according to the guidelines laid down in the Declaration of Helsinki and all procedures involving human subjects were approved by both the Human Research Ethics Committees of the North-West University, Potchefstroom (NWU-00186-15-A1 and NWU-00049-16-A1) and the University of the Witwatersrand, Johannesburg (M150968 and M161045). The Gauteng Health Department, City of Johannesburg District Research Committee and Clinical Manager of Rahima Moosa Mother and Child Hospital gave permission to conduct research at the indicated clinical setting.

## Results

### Participant characteristics and birth outcomes

A total of 595 potentially eligible women residing in Johannesburg, South Africa, were recruited and invited to take part in the study of which 313 volunteered (53%) and signed written informed consent. After screening, 63 women were excluded based on inclusion and exclusion criteria. In total, 250 women were enrolled and completed data collection at baseline (<18 weeks of gestation). Of these, 232 completed follow-ups to delivery: eleven women were lost to follow-up and seven had a miscarriage/intra-uterine foetal death. These 18 cases were included in cross-sectional analysis of data at enrolment, thus all 250 women were included when enrolment data were reported. One entry for birth data was excluded in regression analyses due to early emergency c-section (28 weeks of gestation). There was one maternal fatality shortly after delivery. Several women (n = 29) delivered their babies elsewhere and therefore, birth weight data were missing (see S1 Table). At enrolment there were no significant differences in participant demographic characteristics between women with and without birth weight data (n = 40). There was, however, a significant difference in the anaemia and iron status at enrolment of women with and without birth weight data. Women without birth weight data were significantly more anaemic and had a lower iron status at enrolment than women with birth weight data.

Characteristics of pregnant women at enrolment (<18 weeks of gestation) in the total study group, as well as by birth outcome, are shown in Table 1. Most of the women were of black-African descent (88%) with a median age of 27 (IQR: 24–32) years and gestation of 14 (12–16) weeks at enrolment. A quarter of the women (25%) were born in Zimbabwe, although the majority was born in South Africa (72%). Fifty-eight percent of women have completed secondary school and 23% post-school education. Many women (40%) were unmarried/single and 59% had an LSM score indicative of middle-class living standards. The median BMI (26.3 [23.0–30.6] kg/m^2^) at enrolment was above a healthy range with 33% of women being overweight and 28% obese. More than a quarter of the women were HIV positive (26%). Thirty percent were nulliparous. At enrolment, the only significant difference between women who ultimately delivered premature and non-premature babies were their CRP status. When comparing women who delivered LBW vs non-LBW babies, those who gave birth to LBW babies had a significantly lower BMI at enrolment (24.9 [21.1–26.5] vs 27.1 [23.3–30.8] kg/m^2^) and fewer women had increased CRP (*p<*0.01). The median birth weight was 3050 (2324–3380) grams and 14% (n = 29) of the babies were born with LBW (<2500 g). The median gestational age at birth was 274 (266–282) days and 11% (n = 26) of babies were born preterm (<259 days).

**Table 1:** Characteristics of pregnant women from the NuPED study at enrolment (<18 weeks of gestation) by birth outcome.

	Total sample (n = 250)*	LBW, 14% (n = 29)	Non-LBW, 86% (n = 174)	*p*†	Preterm, 11% (n = 26)	Term, 89% (n = 207)	*p*†
*Characteristics*	*Median (IQR) or n (%)*						
**Age** (years)	27 (24–32)	27 (23–32)	28 (24–32)	0.69	28 (22–34)	27 (24–32)	0.83
**Gestational age** (weeks)	14 (12–16)	14 (11–17)	14 (12–16)	0.99	14 (10–16)	14 (12–16)	0.49
**BMI** (kg/m^2^)	26.3 (23.0–30.6)	24.9 (21.1–26.5)	27.1 (23.3–30.8)	**0.02**	25.3 (21.7–28.4)	26.5 (23.3–30.7)	0.10
Underweight (<18.5 kg/m^2^)	8 (3)	4 (14)	3 (2)	**<0.01**	0	7 (3)	0.26
Normal weight (18.5–24.9 kg/m^2^)	89 (36)	11 (38)	61 (35)		13 (50)	71 (35)	
Overweight (25–29.9 kg/m^2^)	81 (33)	9 (31)	58 (34)		9 (35)	68 (33)	
Obese (≥30 kg/m^2^)	71 (28)	5 (17)	51 (29)		5 (15)	60 (29)	
**Ethnicity**							
Black African	219 (88)	23 (79)	153 (88)	**0.04**	22 (88)	182 (88)	0.94
Mixed ancestry	28 (11)	5 (17)	21 (12)		3 (12)	24 (12)	
White	1 (<1)	-	-		-	-	
Indian	1 (<1)	1 (3)	0		0	1 (1)	
**Country of birth**							
South Africa	172 (72)	23 (85)	117 (70)	0.42	19 (79)	139 (70)	0.73
Zimbabwe	60 (25)	4 (15)	47 (28)		5 (21)	54 (27)	
Lesotho	4 (2)	0	2 (1)		0	4 (2)	
Swaziland	3 (1)	0	1 (1)		0	2 (1)	
**Living Standards Measure (LSM)**							
Low (LSM 1–4)	17 (7)	2 (7)	10 (6)	0.89	1 (4)	14 (7)	0.85
Medium (LSM 5–7)	148 (59)	16 (55)	104 (60)		17(62)	124 (60)	
High (LSM 8–10)	85 (34)	11 (38)	60 (35)		9 (35)	69 (33)	
**Marital status**							
Unmarried/single	100 (40)	18 (62)	66 (38)	0.06	14 (56)	79 (38)	0.36
Married	68 (27)	5 (17)	46 (26)		4 (16)	60 (29)	
Divorced/Separated	2 (1)	1 (3)	1 (1)		0	2 (1)	
Living together	57 (23)	3 (10)	44 (25)		4 (16)	49 (24)	
Traditional marriage^#^[38]	22 (9)	2 (7)	17 (10)		3 (12)	17 (8)	
**Highest level of education**							
Primary school or less	9 (4)	1 (3)	4 (2)	0.94	0	9 (4)	0.28
Grade 8–10	37 (15)	4 (14)	23 (13)		6 (24)	27 (13)	
Grade 11–12	145 (58)	16 (55)	105 (60)		12 (48)	125 (60)	
Post-school education	58 (23)	8 (28)	42 (24)		7 (28)	46 (22)	
**Parity**							
Nulliparous	74 (30)	14 (48)	47 (27)	0.07	10 (39)	57 (28)	0.66
Primiparous	88 (35)	5 (17)	65 (37)		7 (27)	76 (37)	
Multiparous	88 (35)	10 (35)	62 (36)		9 (36)	73 (36)	
**HIV status**							
Positive	64 (26)	6 (21)	43 (25)	0.64	7 (27)	53 (26)	0.89
Negative	186 (74)	23 (79)	131 (75)		19 (73)	154 (74)	
**Inflammatory status**							
Elevated CRP (>5 mg/L)	149 (60)	10 (35)	110 (63)	**<0.01**	10 (39)	130 (63)	**0.02**
Elevated AGP (>1 g/L)	28 (11)	3 (10)	22 (13)	0.73	3 (12)	24 (12)	0.99

LBW: Low birth weight; IQR: interquartile range; CRP: C-reactive protein; AGP: α_1_-acid glycoprotein; LSM: Living Standards Measure

Data are presented as n (%) for categorical variables and median (IQR) for continuous variables.

**†** Mann-Whitney-U test for continuous variables, and Chi-square test for categorical variables.

* n-values are equal to 250 for LSM, highest level of education, parity and HIV status; 239 for Country of birth; and 249 for all other variables.

#: Traditional marriage, recognised under South African customary law, is entered between parties based on tradition which does not require the approval of an officiator for validation. It is also different from civil marriage in that a polygamous marriage is permissible.

### Maternal dietary and supplemented iron intake

The results on dietary iron intake at early pregnancy and supplemented iron intake throughout pregnancy are displayed in Table 2. Median (minimum–maximum range) maternal dietary iron intake as reported at <18 weeks of gestation was 19.1 (4.6–46.1) mg per day from foods, which included fortified foods. There was no significant difference in dietary iron intake between the anaemic and non-anaemic women (*p* = 0.45) nor between the ID and non-ID women (*p* = 0.24). Most women (62%) consumed less iron than the Estimated Average Requirement (EAR) (22 mg/day) for women during pregnancy, and two women (1%) had a dietary iron intake above the upper limit (UL) (45 mg) from foods [39]. The estimated median percentage compliance to routine iron supplementation during the course of pregnancy was 100% (0–100), and the median supplemented iron intake of 55 (0–110) mg/day is reflective of routine iron supplementation. Women reported to buy nutritional supplements in addition to routine iron supplementation which is reflected in the maximum value of total supplemental iron intake of 125 mg elemental iron per day. There was no significant difference in mean supplemental iron intake or supplementation compliance between the anaemic and non-anaemic women (*p* = 0.74 and *p* = 0.83); while there was a significant difference in reported routine supplement intake between the ID and non-ID (*p* = 0.04) women at 36 weeks of gestation.

**Table 2:** Dietary iron intake at early pregnancy (<18 weeks of gestation) and supplemental iron intake throughout pregnancy.

	All women		Anaemic		Non-anaemic			ID		Non-ID		
	n (%)	Median (Min-Max)	n (%)	Median (Min-Max)	n (%)	Median (Min-Max)	p*	n (%)	Median (Min-Max)	n (%)	Median (Min-Max)	p*
Dietary iron intake at early pregnancy, mg/day	250	19.1 (4.6–46.1)	70	19.5 (6.9–43.2)	173	18.7 (4.6–46.1)	0.45	37	19.5 (8.6–39.8)	213	18.9 (4.6–46.1)	0.24
< EAR (<22 mg/d)	155 (62)		41 (59)		111 (64)			23 (62)		132 (32)		
Between EAR and UL	93 (37)		29 (41)		60 (35)			14 (38)		79 (37)		
>UL (>45 mg/d)	2 (1)		0		2 (1)			0		2 (1)		
Reported supplemental iron intake at 36 weeks of gestation:												
Routine supplemental iron intake, mg/day	227	55 (0–110)	63	55 (0–110)	158	55 (0–110)	0.74	34	55 (0–55)	193	55 (0–110)	**0.04**
% Compliance	227	100 (0–100)	63	100 (0–100)	158	100 (0–100)	0.83	34	100 (0–100)	193	100 (0–100)	0.06
Total supplemental iron intake (routine + store-bought) mg/day	227	55 (0–125)	63	55 (8–110)	158	55 (8–125)	0.96	32	55 (8–73)	190	55 (8–125)	0.07

EAR: Estimated Average Requirement; UL: Tolerable Upper Intake Level

Anaemic: Haemoglobin <11 g/dL; ID: Ferritin <15 μg/L

Data are presented as median (minimum-maximum) for continuous variables and n (%) for categorical variables.

* Nonparametric tests for independent samples were used for comparing continuous variables.

### Haematological outcomes with pregnancy progression

**Fig 1:**
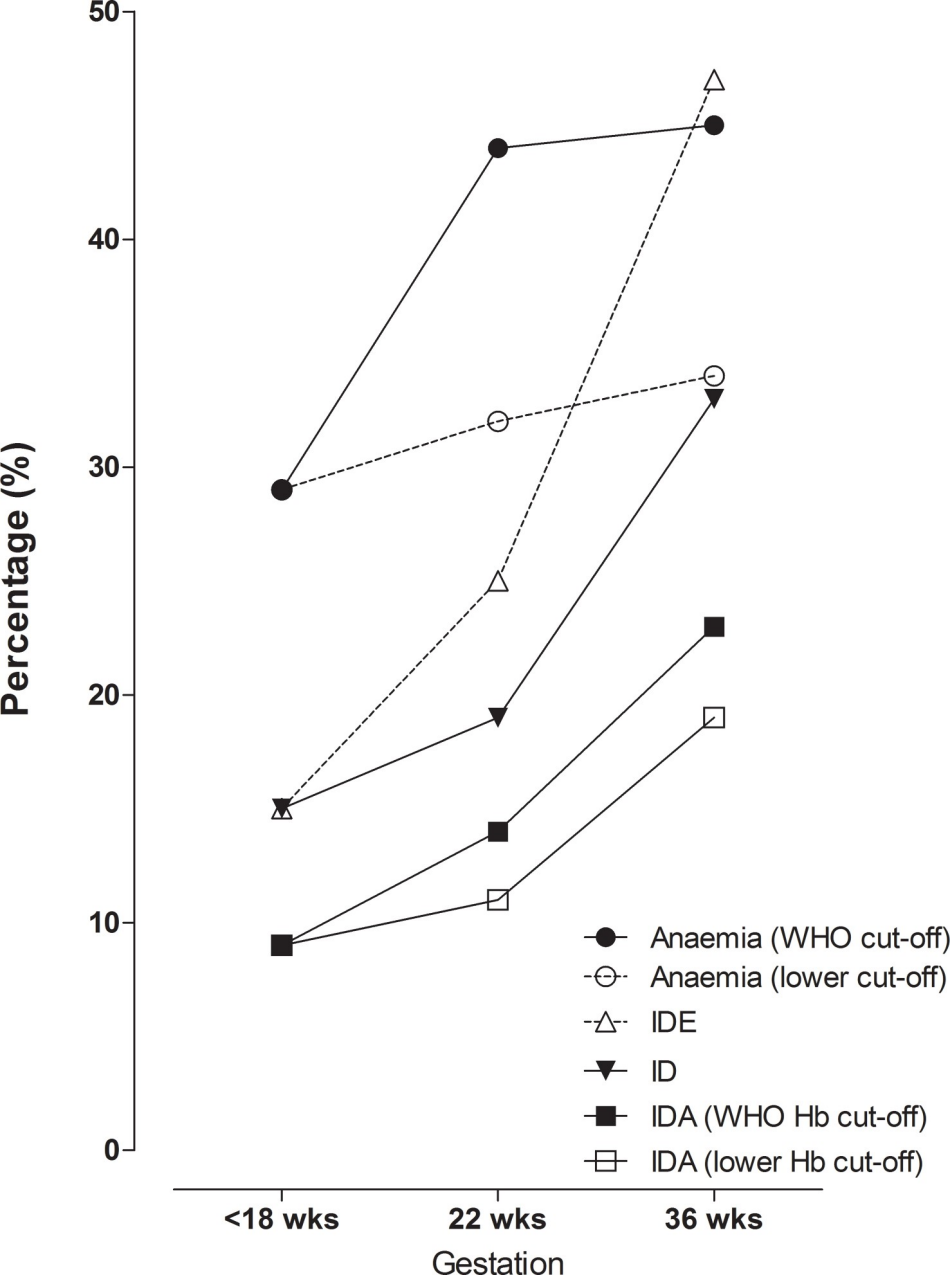
Iron and anaemia status trajectory over the course of pregnancy in women residing in Johannesburg, South Africa. ID–iron depletion; IDE–iron deficiency erythropoiesis; IDA–iron deficiency anaemia; Hb–haemoglobin; wks—weeks. Anaemia (WHO cut-off): haemoglobin <11 g/dL); Anaemia (lower cut-off): haemoglobin <10.5 g/dL at 22 and 36 weeks of gestation; IDE: sTfR >8.3 mg/L ID: Ferritin <15 μg/L; IDA (WHO Hb cut-off): ferritin <15 μg/L plus haemoglobin <11 g/dL; IDA (lower Hb cut-off): ferritin <15 μg/L plus haemoglobin <10.5 g/dL at 22 and 36 weeks of gestation.

Table 3 shows the haematological biomarkers relevant to iron and inflammatory status with pregnancy progression as measured at the three time points. Fig 1 illustrates the anaemia, ID, IDE and IDA trajectory with pregnancy progression. It should be noted that even though 232 women completed follow-up visits up to birth, only 199 blood samples were available for the 36 weeks visit. This is due to 27 women giving birth prematurely (before this visit) and six samples not available for analyses. At enrolment, there were only 3 cases with severe anaemia (haemoglobin <7 g/dL). The median haemoglobin concentration at early pregnancy was 11.7 (10.8–12.7) g/dL and declined to 11.2 (10.1–12.1) g/dL at the second visit (*p<*0.001) and plateaued to the end of pregnancy (11.2 [10.1–12.1] g/dL) (*p* = 0.88). Anaemia prevalence (using the WHO cut-off: haemoglobin <11 g/dL throughout pregnancy) increased from 29% to 44% (*p*<0.001) and 45% (*p* = 0.99) at the three time points, respectively. However, when using the more conservative haemoglobin cut-off of <10.5 g/dL for the second and third time points, the anaemia prevalence of 29% at early pregnancy remained unchanged at the second (32%; *p* = 0.70) and third (34%; *p* = 0.69) visit. Median adjusted ferritin concentrations declined gradually and significantly from 47.6 (21.3–98.7) μg/L to 31.7 (17.8–58.6) μg/L and 20.8 (13.9–40.1) μg/L at the three time points, respectively. ID prevalence increased from 15% to 19% (*p* = 0.06) and 33% (*p* = 0.001). The prevalence of IDA (using WHO haemoglobin cut-off) increased from 9% to 14% (*p* = 0.08) and 23% (*p* = 0.008), while the prevalence of IDA using the lower haemoglobin cut-off changed from 9% to 11% (*p* = 0.68) and 19% (*p* = 0.01). Median sTfR concentrations increased significantly from 4.8 (3.8–6.6) mg/L at <18 weeks to 6.8 (5.4–8.3) and 8.1 (6.5–10.5) mg/L with pregnancy progression. Consequently, IDE prevalence increased significantly from 15% to 25% and 47% during pregnancy. Both median CRP and AGP concentrations declined significantly with pregnancy progression. At enrolment, 60% of participants had elevated CRP (>5 mg/L) and 11% had elevated AGP (>1 g/L) (Table 1).

**Table 3:** Haematological biomarkers of iron status at three time points in pregnant women residing in Johannesburg, South Africa.

	<18 weeks	22 weeks	36 weeks	*p*†	*p*†
Biomarker	Median (IQR)	Median (IQR)	Median (IQR)	(<18 & 22 weeks)	(22 & 36 weeks)
n	250	232	199		
Haemoglobin, g/dL	11.7 (10.8–12.7)	11.2 (10.1–12.1)	11.2 (10.1–12.1)	**<0.001**	0.88
Serum ferritin, μg/L	47.6 (21.3–98.7)	31.7 (17.8–58.6)	20.8 (13.9–40.1)	**<0.001**	**<0.001**
Serum sTfR, mg/L	4.8 (3.8–6.6)	6.8 (5.4–8.3)	8.1 (6.5–10.5)	**<0.001**	**<0.001**
Serum CRP, mg/L	6.5 (3.1–14.4)	6.6 (3.1–12.8)	4.8 (2.8–9.8)	0.06	**<0.001**
Serum AGP, g/L	0.75 (0.63–0.86)	0.61 (0.53–0.74)	0.55 (0.47–0.65)	**<0.001**	**<0.001**

sTfR: soluble transferrin receptor; CRP: C-reactive protein; AGP: α_1_-acid glycoprotein.

Data are presented as medians (interquartile range [IQR]) for continuous variables

**†** Wilcoxon nonparametric tests were used for comparing continuous variables between <18 and 22 weeks and between 22 and 36 weeks of gestation.

### Associations of iron and anaemia status with birth outcomes

Results from the logistic regression analyses of the associations of maternal iron status with LBW as well as preterm birth as binary outcomes are shown in Table 4. The only significant association found was that women who were IDE at 22 weeks of gestation had 3.6 times the risk of giving birth prematurely (OR: 3.57, 95% CI: 1.24, 10.34) as indicated in the fully adjusted model.

**Table 4:** Associations between maternal iron status and birth outcomes (LBW and premature birth) (binary logistic regression, odds ratios and 95% confidence intervals).

		Associations with LBW*							
		**Model 1** (n = 186)			**Model 2** (n = 186)			**Model 3** (n = 156)		
	**Predictor**	**OR**	**95% CI**	***p***	**OR**	**95% CI**	***p***	**OR**	**95% CI**	***p***
<18 weeks	**Anaemia**, Hb <11 g/dL	0.40	0.10, 1.61	0.20	0.38	0.09, 1.64	0.20	0.33	0.06, 1.79	0.20
	**ID**, Fer <15 μg/L	0.27	0.04, 2.01	0.20	0.25	0.03, 1.93	1.83	0.99	0.13, 7.16	0.98
	**IDE**, sTfR >8.3 mg/L	0.18	0.02, 1.64	0.13	0.15	0.02, 1.36	0.09	0.56	0.05, 6.23	0.64
22 weeks	**Anaemia**, Hb <11 g/dL	0.50	0.15, 1.65	0.26	0.47	0.14, 1.65	0.24	0.59	0.13, 2.67	0.49
	**Anaemia**, Hb <10.5 g/dL	0.39	0.10, 1.62	0.20	0.35	0.08, 1.49	0.15	0.59	0.12, 2.95	0.52
	**ID**, Fer <15 μg/L	0.32	0.05, 1.89	0.21	0.31	0.05, 1.93	0.21	0.35	0.4, 3.29	0.36
	**IDE**, sTfR >8.3 mg/L	0.64	0.15, 2.66	0.53	0.57	0.13, 2.49	0.45	0.89	0.22, 5.65	0.89
36 weeks	**Anaemia**, Hb <11 g/dL	1.04	0.31, 3.49	0.95	1.27	0.36, 4.5	0.71	2.47	0.44, 13.83	0.30
	**Anaemia**, Hb <10.5 g/dL	1.37	0.39, 4.78	0.62	1.82	0.49, 6.83	0.37	1.93	0.36, 10.40	0.45
	**ID**, Fer <15 μg/L	1.52	0.44, 5.24	0.51	1.52	0.43, 5.37	0.51	0.98	0.13, 7.16	0.98
	**IDE**, sTfR >8.3 mg/L	0.93	0.28, 3.06	0.90	0.97	0.28, 3.32	0.96	0.90	0.16, 5.05	0.91
		**Associations with premature birth**^**#**^								
		**Model 1** (n = 187)			**Model 2** (n = 187)			**Model 3** (n = 154)		
<18 weeks	**Anaemia**, Hb <11 g/dL	1.22	0.46, 3.26	0.69	1.21	0.45, 3.23	0.71	1.59	0.50, 5.04	0.43
	**ID**, Fer <15 μg/L	1.81	0.60, 5.54	0.30	1.75	0.56, 5.46	0.33	2.44	0.67, 8.90	0.18
	**IDE**, sTfR >8.3 mg/L	1.10	0.70, 1.73	0.67	0.75	0.20, 2.79	0.67	0.98	2.3, 4.12	0.99
22 weeks	**Anaemia**, Hb <11 g/dL	0.51	0.19, 1.34	0.17	0.48	0.18, 1.30	0.15	0.60	0.20, 1.84	0.37
	**Anaemia**, Hb <10.5 g/dL	0.94	0.35, 2.52	0.89	0.92	0.34, 2.51	0.87	1.07	0.34, 3.30	0.91
	**ID**, Fer <15 μg/L	0.89	0.28, 2.89	0.85	0.88	0.27, 2.86	0.83	1.34	0.37, 4.82	0.66
	**IDE**, sTfR >8.3 mg/L	2.46	0.99, 6.12	0.05	2.46	0.99, 6.14	0.05	3.57	1.24, 10.34	**0.02**
36 weeks	**Anaemia**, Hb <11 g/dL	0.66	0.16, 2.81	0.58	0.62	0.14, 2.74	0.52	0.55	0.09, 3.44	0.52
	**Anaemia**, Hb <10.5 g/dL	1.06	0.25, 4.55	0.94	1.03	0.23, 4.62	0.97	1.06	0.16, 6.92	0.94
	**ID**, Fer <15 μg/L	1.04	0.24, 4.43	0.96	1.01	0.23, 4.39	0.97	0.35	0.04, 3.21	0.35
	**IDE**, sTfR >8.3 mg/L	1.61	0.41, 6.32	0.50	1.58	0.38, 6.58	0.53	2.05	0.38, 11.09	0.40

Hb: haemoglobin; Fer: serum ferritin; LBW: low birth weight; ID: iron depletion; IDE: iron deficiency erythropoiesis; sTfR: soluble transferrin receptor; OR: odds ratio; CI: confidence interval

* Model 1 for low birth weight adjusted for: maternal age, gestational age at birth and sex of the baby; model 2 adjusted in addition to model 1: parity and living standards measure (socio-economic status); model 3 adjusted in addition to models 1 and 2: HIV status, maternal BMI at enrolment and glucose tolerance.

# Model 1 for premature birth adjusted for: maternal age, baby sex and delivery intervention (induction or caesarean section); models 2 and 3 adjusted for the same additional factors as for low birth weight.

*p* values of <0.05 were considered significant.

Table 5 shows the results from the multiple linear regression analyses on the associations of iron and anaemia status with birth weight (in grams) and gestational age at birth (in days) as outcomes. In the fully adjusted model, anaemia (haemoglobin <10.5 g/dL) at 22 weeks of gestation was associated with a 207 g higher birth weight in neonates (β = 207.0; 95% CI: 70.5, 343.6). When anaemia was defined according to the WHO haemoglobin cut-off (<11 g/dL), anaemia was associated with a 135g higher birth weight in neonates (β = 135.4; 95% CI: 4.8, 266.1). Similarly, ID at 22 weeks was associated with a 205g higher birth weight (β = 205.4; 95% CI: 45.6, 365.1). IDE at 36 weeks was associated with a 178 g higher birth weight (β = 178.0; 95% CI: 47.2, 308.7).

**Table 5:** Associations between maternal iron status and birth outcomes (birth weight and gestational age at birth) (multivariable linear regression, β-values and 95% confidence intervals).

		**Birth weight** (n = 203)*								
		**Model 1**			**Model 2**			**Model 3**		
	**Predictor**	**β**	**95% CI**	***P***	**β**	**95% CI**	***P***	**β**	**95% CI**	***P***
<18 weeks	**Anaemia**, Hb <11 g/dL	18.2	-126.1, 162.6	0.80	24.0	-120.2, 168.2	0.74	63.5	-87.4, 214.4	0.41
	**ID**, Fer <15 μg/L	-10.3	-194.3, 173.8	0.91	22.2	-163.2, 207.5	0.81	42.0	-152.1, 236.0	0.67
	**IDE**, sTfR >8.3 mg/L	150.3	-29.3, 326.9	0.10	183.9	3.8, 363.9	0.05	128.7	-60.5, 317.9	0.18
22 weeks	**Anaemia**, Hb <11 g/dL	104.0	-18.1, 226.1	0.09	145.0	21.2, 268.9	**0.02**	135.4	4.8, 266.1	**0.04**
	**Anaemia**, Hb <10.5 g/dL	177.1	48.0, 306.2	**<0.01**	213.7	84.5, 342.8	**<0.01**	207.0	70.5, 343.6	**<0.01**
	**ID**, Fer <15 μg/L	210.2	47.0, 373.3	**0.01**	190.0	38.0, 342.1	**0.02**	205.4	45.6, 365.1	**0.01**
	**IDE**, sTfR >8.3 mg/L	40.1	-109.4, 189.7	0.59	52.3	-97.0. 201.6	0.49	58.1	-97.0, 213.3	0.46
36 weeks	**Anaemia**, Hb <11 g/dL	-45.5	-183.0, 92.1	0.52	-24.9	-166.4, 116.7	0.73	25.6	-120.9, 172.0	0.73
	**Anaemia**, Hb <10.5 g/dL	13.3	-131.8, 158.3	0.86	33.8	-113.6, 181.1	0.65	105.9	-42.8, 254.6	0.16
	**ID**, Fer <15 μg/L	37.6	-97.1, 172.2	0.58	48.9	-85.3, 183.0	0.47	75.7	-61.0, 212.5	0.28
	**IDE**, sTfR >8.3 mg/L	199.2	-5.7, 244.1	**0.06**	164.22	35.0, 293.4	**0.01**	178.0	47.2, 308.7	**<0.01**
		**Gestational age at birth** (n = 233)^#^								
		**Model 1**			**Model 2**			**Model 3**		
<18 weeks	**Anaemia**, Hb <11 g/dL	1.34	-3.20, 5.87	0.56	1.34	-3.24, 5.91	0.57	1.39	-3.65, 6.43	0.59
	**ID**, Fer <15 μg/L	-3.27	-9.03, 2.50	0.27	-3.68	-9.54, 2.18	0.22	-3.40	-9.84, 3.05	0.30
	**IDE**, sTfR >8.3 mg/L	0.66	-504, 6.35	0.82	0.35	-5.43, 6.14	0.90	1.56	-4.80, 7.91	0.63
22 weeks	**Anaemia**, Hb <11 g/dL	4.65	0.73, 8.58	**0.02**	4.57	0.51, 8.63	**0.03**	4.82	0.39, 9.24	**0.03**
	**Anaemia**, Hb <10.5 g/dL	2.23	-2.25, 6.70	0.33	1.98	-2.60, 6.55	0.40	3.07	-1.97, 8.11	0.23
	**ID**, Fer <15 μg/L	0.53	-4.70, 5.76	0.84	0.01	-0.03, 0.04	0.79	1.14	-4.64, 6.91	0.70
	**IDE**, sTfR >8.3 mg/L	-2.45	-7.16, 2.26	0.31	-2.56	-7.31, 2.19	0.29	-2.90	-8.07, 2.28	0.27
36 weeks	**Anaemia**, Hb <11 g/dL	-0.49	-4.88, 3.89	0.82	-0.70	-5.26, 3.85	0.76	2.50	-5.33, 4.50	0.87
	**Anaemia**, Hb <10.5 g/dL	0.07	-4.55, 4.68	0.98	-0.08	-4.82, 4.66	0.97	-0.80	-5.83, 4.22	0.75
	**ID**, Fer < 15 μg/L	2.56	-2.03, 7.16	0.27	2.50	-2.15, 7.14	0.29	2.26	-2.61, 7.12	0.36
	**IDE**, sTfR >8.3 mg/L	-1.01	-5.30, 3.30	0.64	-1.36	-5.90, 3.17	0.55	-0.33	-5.11, 4.46	0.89

Hb: haemoglobin; Fer: serum ferritin; ID: iron depletion; IDE: iron deficiency erythropoiesis; sTfR: soluble transferrin receptor; CRP: C-reactive protein; AGP: α_1_-acid glycoprotein; CI: confidence interval

* Model 1 for birth weight adjusted for: maternal age, gestational age at birth and sex of the baby; model 2 adjusted in addition to model 1: parity and living standards measure (socio-economic status); model 3 adjusted in addition to models 1 and 2: HIV status, maternal BMI at enrolment and glucose tolerance.

# Model 1 for gestational age at birth adjusted for: maternal age, baby sex and delivery intervention (induction or caesarean section); models 2 and 3 adjusted for the same additional factors as for birth weight.

*p* values of <0.05 were considered significant.

Anaemia defined using the WHO cut-off (haemoglobin <11 g/dL) at 22 weeks of gestation was associated with an increase in gestational age by 5 days (β = 4.82; 95% CI: 0.39, 9.24). This association did not hold when the lower haemoglobin cut-off was used (haemoglobin <10.5 g/dL).

To further investigate the association of low and high concentrations of iron biomarkers with birth outcomes, haematological biomarker concentrations were divided in quartiles, and univariate comparisons of birth weight and gestational age conducted in adjusted models as shown in Table 6. There was a significant difference between the second lowest (haemoglobin: 10.8–11.7 g/dL) and the highest quartile (haemoglobin >12.7 g/dL) of haemoglobin at <18 weeks of gestation with gestational age at birth. Women in the second lowest haemoglobin quartile at early pregnancy had a significantly shorter gestation by 7 days (β = -6.9, 95% CI: -13.3, -0.6) when compared to women in the highest quartile. In addition, we found significant differences in birth weight between quartiles of serum ferritin concentrations at 22 weeks and 36 weeks of gestation. Women in the lowest ferritin quartile (<17.8 μg/L) at 22 weeks gave birth to babies weighing 311.8 g (95% CI: 94.8, 528.8) more than those in the highest quartile (>58.6 μg/L). At 36 weeks of gestation, those in the lowest ferritin quartile (ferritin <13.85 μg/L) gave birth to babies weighing 410.3 g (95% CI: 184.3, 636.3) more than those in the highest quartile (ferritin >40.08 μg/L) (*p*<0.001). Women in the third ferritin quartile (ferritin 20.80–40.08 μg/L) at 36 weeks of gestation also had significantly heavier babies (276.2 g, 95% CI: 72.2, 480.2) than women in the highest quartile (>40.08 μg/L).

**Table 6:** Associations between quartiles of haemoglobin, ferritin and sTfR at three time points with birth weight and gestational age at birth (β-values and 95% confidence intervals).

Iron index	Median birth weight* (g)	n	β (95% CI)	*p*	Median gestational age^#^ (days)	n	β (95% CI)	*p*
	**<18 weeks**				**<18 weeks**			
Haemoglobin (g/dL)				0.28				0.15
Quartile 1: <10.8	3058	35	-39.7 (-258.5, 179.1)	0.72	271	36	-3.7 (-10.3, 2.9)	0.27
Quartile 2: 10.8–11.7	3122	42	23.6 (-187.2, 234.4)	0.83	268	41	-6.9 (-13.3, -0.6)	**0.03**
Quartile 3: 11.8–12.7	2923	36	-174.4 (-392.0, 43.1)	0.12	273	37	-1.3 (-7.9, 5.3)	0.70
Quartile 4: >12.7	3098	40	1		275	39	1	
Ferritin (μg/L)				0.57				0.07
Quartile 1: <21.30	3151	37	123.2 (-93.4, 339.87)	0.26	267	38	-6.0 (-12.2, 0.25)	0.06
Quartile 2: 21.30–47.56	3019	43	-8.14 (-212.4, 196.1)	0.94	276	43	2.5 (-3.5, 8.5)	0.41
Quartile 3: 47.57–98.68	3079	34	52.1 (-163.5, 267.7)	0.63	273	33	-0.5 (-6.9, 5.9)	0.88
Quartile 4: >98.68	3027	42	1		273	42	1	
sTfR (mg/L)				0.64				0.99
Quartile 1: >6.63	3161	41	130.7 (-81.4, 342.8)	0.23	273	42	0.1 (-6.3, 6.4)	0.99
Quartile 2: 4.82–6.63	3094	39	29.6 (-189.4, 248.6)	0.79	272	38	0.6 (-6.0, 73)	0.85
Quartile 3: 3.76–4.81	3035	36	41.5 (-177.1, 260.4)	0.71	270	36	-0.3 (-6.9, 6.3)	0.92
Quartile 4: <3.76	2967	40	1		274	40	1	
	**22 weeks**				**22 weeks**			
Haemoglobin (g/dL)				0.52				0.72
Quartile 1: <10.8	3163	38	137.9 (-79.7, 355.42)	0.21	274	40	2.4 (-4.2, 9.0)	0.47
Quartile 2: 10.8–11.2	3068	41	12.6 (-171.9, 257.1)	0.70	272	41	0.4 (-6.1, 6.8)	0.91
Quartile 3:11.3–12.1	3007	36	-18.17 (-229.4, 193.1)	0.87	270	35	-1.5 (-7.9, 4.9)	0.64
Quartile 4: >12.1	3025	41	1		272	40	1	
Ferritin (μg/L)				**0.045**				0.60
Quartile 1: <17.78	3248	33	311.8 (94.8, 528.8)	**0.005**	270	35	-4.2 (-10.8, 2.5)	0.22
Quartile 2: 17.78–31.66	3041	41	104.8 (-104.0, 313.6)	0.32	271	41	-3.6 (-10.1, 2.8)	0.27
Quartile 3: 31.67–58.63	3067	40	131.2 (-73.8, 336.1)	0.21	272	39	-2.9 (-9.2, 3.5)	0.37
Quartile 4: >58.63	2936	40	1		275	39	1	
sTfR (mg/L)				0.35				0.37
Quartile 1: >8.33	2962	36	-109.2 (-330.1, 111.8)	0.33	268	37	-4.7 (-11.3, 1.9)	0.10
Quartile 2: 6.85–8.33	3044	39	-180.6 (-394.5, 33.33)	0.10	273	39	0.6 (-5.9, 7.1)	0.86
Quartile 3: 5.36–6.84	3025	42	-156.2 (-366.8, 54.34)	0.15	273	41	-1.0 (-7.01, 0.2)	0.76
Quartile 4: <5.36	3231	37			275	37	1	
	**36 weeks**				**36 weeks**			
Haemoglobin (g/dL)				0.99				0.75
Quartile 1: <10.03	3169	31	-0.6 (-242.4, 241.2)	1.00	275	32	-2.1 (-7.2, 3.0)	0.41
Quartile 2: 10.03–11.2	3140	36	-29.0 (-255.1, 197.1)	0.80	275	34	-2.1 (-7.0, 2.8)	0.40
Quartile 3:11.3–12.1	3143	35	-26.1 (-246.4, 194.3)	0.82	274	35	-2.3 (-7.0, 2.4)	0.34
Quartile 4: >12.1	3169	39	1		277	39	1	
Ferritin (μg/L)				**0.004**				0.57
Quartile 1: <13.85	3346	30	410.3 (184.3, 636.3)	**<0.001**	277	31	1.4 (-3.7, 6.5)	0.58
Quartile 2: 13.85–20.79	3129	33	193.7 (-20.7, 408.0)	0.08	273	32	-2.1 (-7.0, 2.7)	0.39
Quartile 3: 20.80–40.08	3212	46	276.2 (72.2, 480.2)	**0.008**	275	46	-0.6 (-5.2, 3.9)	0.79
Quartile 4: >40.08	2936	35	1		275	34	1	
sTfR (mg/L)				0.17				0.84
Quartile 1: >10.53	3300	30	156.1 (-71.8, 384.1)	0.18	275	30	0.8 (-4.2, 5.8)	0.75
Quartile 2: 8.06–10.53	3096	36	-60.1 (-272.3, 152.1)	0.58	274	35	-1.4 (-6.0, 3.2)	0.56
Quartile 3: 6.45–8.05	3102	41	-78.0 (-283.7, 127.6)	0.45	275	41	-0.3 (-4.8, 4.1)	0.88
Quartile 4: <6.45	3149	37	1		276	37	1	

*Birth weight adjusted for maternal age, gestational age at birth, sex of the baby, parity, living standards measure (socio-economic status); HIV status, maternal BMI at enrolment and glucose tolerance.

#Gestational age adjusted for maternal age, delivery intervention, sex of the baby, parity, living standards measure (socio-economic status); HIV status, maternal BMI at enrolment and glucose tolerance

## Discussion

In this prospective study of pregnant women residing in Johannesburg, South Africa, the prevalence of anaemia, ID and IDE increased despite iron supplementation forming part of routine antenatal care. We found that ID and anaemia at mid-pregnancy, as well as IDE at late-pregnancy were associated with higher birth weight. In contrast, women with IDE at mid-pregnancy had a 3.6 times higher risk of giving birth prematurely and women with a lower haemoglobin at early pregnancy gave birth significantly earlier than those in the highest haemoglobin quartile.

In this sample of generally healthy, non-smoking, singleton pregnancies from an urban area of South Africa, we observed a similar iron deficiency prevalence during early pregnancy as in women of reproductive age participating in previous national surveys [7,8,40]. More than a quarter (29%) of the women were anaemic at early pregnancy, while 15%, 15% and 9% of women were ID, IDE and IDA, respectively. The WHO recommends routine daily iron supplementation (30–60 mg elemental iron) plus folic acid for all pregnant women to cover increased iron requirements. Furthermore, in settings where at least 40% of pregnant women have haemoglobin concentrations <11 g/dL, a daily dose of 60 mg elemental iron should be preferred over a lower dose [1,41,42]. Even though the prevalence of anaemia is less than 40% in South African women of reproductive age, the recommendation is to supplement all pregnant women with 60 mg elemental iron daily (in conjunction with folic acid and calcium) [11]. In our setting, all pregnant women receive 55 mg elemental iron (170 mg dried ferrous sulphate) daily with folic acid and calcium. Compliance to routine supplementation was high in our study (median of 100%) and some women reported purchasing supplements from shops in addition to the routine regime. Even so, we found significant declines in iron status with pregnancy progression. Two cohort studies in West African countries with routine iron supplementation for pregnant women observed a similar decline in iron status during pregnancy [43,44]. The sharp decline in haemoglobin concentrations that we observed in our sample of women at mid-pregnancy can be explained by maternal red blood cell mass and plasma volume expansion leading to haemodilution [45].

It is also known that serum ferritin concentrations gradually decline with pregnancy progression. While haemodilution may explain this phenomenon, it has been suggested that declines in serum ferritin concentrations may reflect iron mobilisation from stores to cover increased requirements for red blood cell production, as well as placental transfer to the foetus [46]. In our sample of pregnant women, 47% had elevated sTfR concentrations by late pregnancy. It is unclear why these women receiving iron supplements experienced such a marked increase in sTfR concentrations. Increased sTfR expression is reflective of erythropoietic activity, typically expected with red blood cell mass expansion in pregnancy [47]. In addition to increased need, the women in this study showed significant reductions in ferritin concentrations with pregnancy progression and depleted iron stores could be one reason for increased sTfR [48]. However, increased sTfR is also strongly associated with functional tissue iron deficiency, indicating that iron cannot be mobilised for erythropoiesis despite adequate iron stores [49]. Elevated concentrations of the hormone hepcidin, which is the main regulator of systemic iron homeostasis [46], may explain this observation. With sufficient systemic iron (thus in iron-replete cases), hepcidin concentrations increase, which in turn reduce the release of iron from enterocytes, macrophages and hepatocytes. Conversely, production of hepcidin is suppressed during iron deficiency to allow release of iron from stores and to increase dietary iron absorption. Recent studies showed that hepcidin is actively reduced during the second and third trimesters of pregnancy to support increased iron requirements [44]. However, hepatocyte hepcidin production increases with inflammation irrespective of iron status [46]. More than half of the women enrolled in our study (n = 149; 60%) entered the study with elevated CRP (>5 mg/L) concentrations, indicating a high prevalence of acute and sub-clinical inflammation. African ethnicity has been associated with higher circulating CRP concentrations [50]. In addition, this sample of pregnant women had a high prevalence of overweight and obesity (33% and 28%, respectively), as well as HIV infection (26%), which may have contributed to a more inflammatory state. These factors may have led to an increase in hepcidin concentrations, and consequently to a reduction in iron absorption and release from hepatic stores. In addition, high intakes of calcium (1 g calcium supplementation/day to all pregnant women in South Africa) have been shown to inhibit iron absorption [51]. The *Guidelines for maternity care South Africa* [11] indicates that calcium “is best taken 4 hours before or after iron supplements”. However, it is not known how well this recommendation is implemented. This context may explain the increasing prevalence of ID, IDE and IDA with pregnancy progression in an iron supplemented population.

In our sample, the mean daily iron intake (19 mg, 4.6–46.1 mg) at early pregnancy was approximately 3 mg less than the EAR for pregnant women (22 mg/day), but sufficient to meet the EAR for non-pregnant women (8.1 mg/day) [39]. This supports the current recommendation that iron should be supplemented during pregnancy, but arguably at a lower dose in this setting. Women’s iron requirements differ depending on stage of pregnancy. Prior to pregnancy, women’s iron requirements are higher than for men due to menstruation. In the first trimester of pregnancy iron requirements are less than prior to pregnancy due to cessation of menses [46], while requirements increase drastically from the second trimester due to blood volume expansion and increased erythropoietic activity. Therefore, if reported dietary intakes during early pregnancy are reflective of dietary intakes prior to pregnancy, it is likely that most women achieved the recommended iron intake prior to pregnancy. This may explain the relatively low prevalence of ID at enrolment and highlights the need for exploring other determinants of anaemia [52] in women, such as other micronutrient deficiencies and/or inflammation.

In our sample, anaemia and ID at mid-pregnancy were associated with a 207 g and 205 g higher birth weight, respectively. Consistently, IDE at late pregnancy was associated with a 178 g higher birth weight. When comparing quartiles of ferritin concentration at mid-pregnancy, women in the lowest quartile (ferritin <17.78 μg/L) gave birth to significantly heavier babies (312 g) than women in the highest quartile (ferritin >58.63 μg/L). To our knowledge, we are the second study in an African setting (with routine iron supplementation [43]) to find this association. In the cohort of pregnant women in Papua New Guinea (n = 279), malaria infection was common and the prevalence of anaemia (haemoglobin <11 g/dL) at enrolment (±25 weeks of gestation) very high (95%). Lower ferritin concentrations at enrolment were associated with higher mean birth weights, and iron deficient women gave birth to 230 g heavier newborns when compared to iron-replete women. The authors indicated that only 7% and 12% of the association was mediated through placental and peripheral malaria infection, respectively, demonstrating an association between iron deficiency and higher birth weight through Malaria-independent mechanisms. Similar associations have been found elsewhere. A Chinese cohort study (n = 511) of non-anaemic pregnant women receiving iron supplements as part of routine antenatal care also found a significantly higher birth weight in the lowest compared to the highest ferritin quartile [53]. Furthermore, an Indian cohort (n = 1196) (non-anaemic with supplementation) showed similar results with the highest tertile of supplemental iron intake associated with low birth weight [54].

The observed inverse associations between maternal iron status and birth weight in settings of routine iron supplementation can be interpreted from two viewpoints. Firstly, the association between ID, anaemia and IDE with higher birth weight could be an indication that antenatal iron supplementation is protective in iron depleted women, resulting in improved foetal growth [55]. Systematic reviews on the efficacy of antenatal iron supplementation versus placebo have shown significant reductions in anaemia and iron deficiency at term. Evidence for a beneficial effect on birth outcomes is, however, less clear [56–58]. The most recent Cochrane review indicated that there is low quality evidence for iron supplementation reducing the risk for low birth weight (RR 0.84; 95% CI: 0.69, 1.03; 11 studies) [57]. In contrast, there is emerging evidence of a U-shape association of iron status and haemoglobin with birth weight [5,6]. Thus, the second viewpoint is from the right side of this U-shape association, i.e. high iron intakes being associated with lower birth weight. Routine iron supplementation in a mixed population of deficient and replete women may contribute to mixed results. In our sample, iron supplementation may have had a negative impact on foetal growth in iron-replete women. Hwang and colleagues [59] found that excessive maternal iron intake at mid-pregnancy was associated with reduced foetal growth in a South Korean cohort (n = 337). The foetuses of women in the third tertile of total iron intake had smaller outcomes in biparietal diameter, abdominal circumference and femur length at mid-pregnancy. Even though this South Korean cohort demonstrated lower than recommended iron intake from foods, supplementation contributed to intakes above the Tolerable Upper Intake Level (45 mg). Other studies indicate that iron supplementation in iron-replete women is associated with adverse maternal and foetal outcomes [54,60], although results are inconsistent [61]. When considering the median ferritin and haemoglobin concentrations as well as the prevalence of ID and anaemia at early pregnancy in our sample, it is apparent that most women were iron-replete while receiving iron supplementation. We speculate that provision of supplemental iron in these iron-replete women may have had negative consequences to the mother, which may have compromised foetal growth. These negative consequences may include oxidative damage and haemoconcentration which consequently result in impaired placental perfusion and thus foetal growth [58,62]. There is evidence that unabsorbed supplemental iron reaching the colon can negatively alter gut microbiome composition and increase gut inflammation [63]. This supports the notion that strategies to prevent iron deficiency during pregnancy should consider the inflammation burden in the target population, and take relevant actions to reduce inflammation in an effort to improve iron absorption and utilisation.

When we assessed the predictors for gestational age, associations were different than for birth weight. Firstly, lower haemoglobin in early pregnancy was associated with shorter gestation which supports previous research [64]. It is suspected that these women entered pregnancy with anaemia since the quartiles associated with the shorter gestation reflects pre-pregnancy anaemia, i.e. haemoglobin <12 g/dL, and haemodilution only peaks late in second trimester or early third trimester [45]. Secondly, as expected, poor iron status increased the risk for premature birth. IDE at mid-pregnancy quadrupled the risk for premature birth. It would be expected that in a supplemented sample the risk would be attenuated in the ID group [57], however, as explained above, elevated sTfR concentrations could be a consequence of inflammation causing iron not to be mobilised.

The key strength of this study was that data were collected prospectively with multiple variables and data collection points across pregnancy. In addition, the iron biomarkers assessed allowed for a complete description of iron status. Previous studies may not have considered concomitant inflammation when interpreting iron status data, however, our analyses included CRP and AGP, which were used to adjust ferritin concentrations accordingly [29]. The analyses were strengthened due to assessment and inclusion of several confounders.

The study limitations should be considered when interpreting the results. Due to the observational study design, conclusions on causality of observed relationships are not possible. However, our findings may generate hypotheses for further investigation, given the consistency of results. An additional limitation is self-selection bias at recruitment since women were recruited at primary healthcare clinics and then volunteered and agreed to participate at a different setting. Lost to follow-up resulted in missing birth weight data, which may have skewed results. Lastly, the sample was of relatively small size and not representative of the general population. Women were selected to be non-smoking, generally healthy and presented at primary healthcare clinics somewhat earlier than the typical 20 weeks of gestation [65].

In conclusion, we found an increase in ID, IDE and IDA with pregnancy progression despite routine iron supplementation in an urban South African setting. We observed an inverse association between maternal iron status and birth weight, while IDE at mid-pregnancy increased the risk for premature birth. These results add to the raising concern on the consequences of iron supplementation in iron-replete pregnant women. Nonetheless, there is no question that ID and anaemia should be prevented in pregnancy. However, the challenge remains on how to do so safely in a public health setting. Considering that South Africa has a well-implemented food fortification programme, high prevalence of inflammation, possible influence of antenatal calcium supplements on iron absorption, as well as the known risks associated with both low and high iron exposure, we recommend that the current antenatal supplementation regime in South Africa be revisited.

## Supporting information

S1 Table Characteristics and iron status of pregnant women from the NuPED study at enrolment (<18 weeks of gestation) by birth weight data availability^¥^. IQR: interquartile range; CRP: C-reactive protein; AGP: α_1_-acid glycoprotein; LSM: Living Standards Measure; Hb: Haemoglobin; Fer: ferritin; sTfR: soluble transferrin receptor.
Data are presented as n (%) for categorical variables and median (IQR) for continuous variables.
¥ Women who had a miscarriage or intrauterine foetal death (IUFD) also had missing birth weight data (n = 7), but were not considered in this table.
**†** Mann-Whitney-U test for continuous variables, and Chi-square test for categorical variables.
* n-values are equal to 250 for LSM, highest level of education, parity, HIV status, iron stores and iron deficiency erythropoiesis; 243 for anaemia; 239 for Country of birth; and 249 for all other variables.
#: Traditional marriage, recognised under South African customary law, is entered between parties based on tradition which does not require the approval of an officiator for validation. It is also different from civil marriage in that a polygamous marriage is permissible.
(DOCX)

## References

[1] World Health Organization. Nutritional anaemias: tools for effective prevention and control [Internet]. Geneva, Switzerland: World Health Organization; 2017 Available: https://www.who.int/nutrition/publications/micronutrients/anaemias-tools-prevention-control/en/

[2] Darnton-Hill I, Mkparu UC. Micronutrients in pregnancy in low- and middle-income countries. Nutrients. 2015;7: 1744–1768. 10.3390/nu7031744 25763532 PMC4377879

[3] Cetin I, Berti C, Calabrese S. Role of micronutrients in the periconceptional period. Hum Reprod Update. 2009;16: 80–95. 10.1093/humupd/dmp025 19567449

[4] Chang S, Zeng L, Brouwer ID, Kok FJ, Yan H. Effect of Iron Deficiency Anemia in Pregnancy on Child Mental Development in Rural China. Pediatrics. 2013;131: e755–e763. 10.1542/peds.2011-3513 23400604

[5] Brannon PM, Taylor CL. Iron supplementation during pregnancy and infancy: uncertainties and implications for research and policy. Nutrients. 2017;9: 1327 10.3390/nu9121327 29210994 PMC5748777

[6] Dewey KG, Oaks BM. U-shaped curve for risk associated with maternal hemoglobin, iron status, or iron supplementation. Am J Clin Nutr. 2017;106: 1694S–1702S. 10.3945/ajcn.117.156075 29070565 PMC5701708

[7] National Department of Health (NDoH), Statistics South Africa (Stats SA), South African Medical Research Council (SAMRC), ICF. South Africa Demographic and Health Survey 2016. Pretoria, South Africa and Rockville, Maryland, USA; 2019.

[8] Shisana O, Labadarios D, Rehle T, Simbayi L, Zuma K, Dhansay A, et al South African National Health and Nutrition Examination Survey, 2012 (SANHANES-1) [Internet]. 2nd ed Cape Town: HSRC Press; 2014 Available: http://www.hsrc.ac.za/en/research-data/view/6493

[9] Harika R, Faber M, Samuel F, Kimiywe J, Mulugeta A, Eilander A. Micronutrient status and dietary intake of iron, vitamin A, iodine, folate and zinc in women of reproductive age and pregnant women in Ethiopia, Kenya, Nigeria and South Africa: a systematic review of data from 2005 to 2015. Nutrients. 2017;9: 1096 10.3390/nu9101096 28981457 PMC5691713

[10] Steyn NP, Wolmarans P, Nel JH, Bourne LT. National fortification of staple foods can make a significant contribution to micronutrient intake of South African adults. Public Health Nutr. 2008;11: 307–313. 10.1017/S136898000700033X 17610752

[11] National Department of Health. Guidelines for maternity care in South Africa [Internet]. Pretoria, South Africa; 2015. Available: https://www.health-e.org.za/2015/11/17/guidelines-maternity-care-in-south-africa/

[12] Tunkyi K, Moodley J. Prevalence of anaemia in pregnancy in a regional health facility in South Africa. South African Med J. 2015;106: 101 10.7196/SAMJ.2016.v106i1.9860 26792317

[13] Mwangi MN, Prentice AM, Verhoef H. Safety and benefits of antenatal oral iron supplementation in low-income countries: a review. Br J Haematol. 2017;177: 884–895. 10.1111/bjh.14584 28272734 PMC5485170

[14] Symington EA, Baumgartner J, Malan L, Zandberg L, Ricci C, Smuts CM. Nutrition during pregnancy and early development (NuPED) in urban South Africa: a study protocol for a prospective cohort. BMC Pregnancy Childbirth. BMC Pregnancy and Childbirth; 2018;18: 308 10.1186/s12884-018-1943-6 30041623 PMC6056931

[15] Shisana O, Rehle T, Simbayi LC, Zuma K, Jooste S, Zungu N, et al South African national HIV prevalence, incidence and behaviour survey, 2012 [Internet]. Cape Town: HSRC Press; 2014 Available: http://repository.hsrc.ac.za/handle/20.500.11910/2490

[16] Johnson TS, Engstrom JL, Gelhar DK. Intra- and interexaminer reliability of anthropometric measurements of term infants. J Pediatr Gastroenterol Nutr. 1997;24: 497–505. 10.1097/00005176-199705000-00001 9161941

[17] United Nations Children’s Fund, World Health Organization. Low birthweight: country, regional and global estimates [Internet]. New York; 2004. Available: http://whqlibdoc.who.int/publications/2004/9280638327.pdf

[18] The American College of Obstetricians and Gynecologists’ Committee on Obstetric Practice. Methods for estimating the due date. Obstet Gynecol. 2017;129: 959–960. 10.1097/AOG.0000000000002038

[19] March of Dimes, PMNCH, Save the Children, World Health Organization (WHO). Born Too Soon: The gobal action report on preterm birth. Howson C, Kinney M, Lawn J, editors. Geneva: World Health Organization; 2012.

[20] Shim J-S, Oh K, Kim HC. Dietary assessment methods in epidemiologic studies. Epidemiol Health. 2014;36: e2014009 10.4178/epih/e2014009 25078382 PMC4154347

[21] MacIntyre UE, Venter CS, Vorster HH, Steyn HS. A combination of statistical methods for the analysis of the relative validation data of the quantitative food frequency questionnaire used in the THUSA study. Public Health Nutr. 2000;4: 45–51. 10.1079/PHN200039 11315680

[22] MacIntyre UE, Kruger HS, Venter CS, Vorster HH. Dietary intakes of an African population in different stages of transition in the North West Province, South Africa: The THUSA study. Nutr Res. 2002;22: 239–256. 10.1016/S0271-5317(01)00392-X

[23] Wentzel-Viljoen E, Laubscher R, Kruger A. Using different approaches to assess the reproducibility of a culturally sensitive quantified food frequency questionnaire. South African J Clin Nutr. 2011;24: 143–148. Available: http://www.ajol.info/index.php/sajcn/article/view/69592

[24] Wolmarans P, Danster N, Dalton A, Rossouw K, Schönfeldt H. Condensed Food Composition Tables for South Africa. Cape Town: Medical Research Council; 2010.

[25] Langenhoven M, Conradie P, Wolmarans P, Faber M. MRC Food Quantities Manual. Parow: South African Medical Research Council; 1991.

[26] Pavord S, Myers B, Robinson S, Allard S, Strong J, Oppenheimer C. UK guidelines on the management of iron deficiency in pregnancy. Br J Haematol. 2012;156: 588–600. 10.1111/j.1365-2141.2011.09012.x 22512001

[27] South Australian Maternal & Neonatal Community. Clinical Guideline Anaemia in Pregnancy. J South Aust Perinat Pract Guidel. 2016; 3–20. 10.7326/M14-1333

[28] Brindle E, Lillis L, Barney R, Hess SY, Wessells KR, Ouédraogo CT, et al Simultaneous assessment of iodine, iron, vitamin A, malarial antigenemia, and inflammation status biomarkers via a multiplex immunoassay method on a population of pregnant women from Niger. PLoS One. 2017;12: 1–20. 10.1371/journal.pone.0185868 28982133 PMC5628875

[29] Thurnham DI, Northrop-Clewes CA, Knowles J. The use of adjustment factors to address the impact of inflammation on vitamin A and iron status in humans. J Nutr. 2015;145: 1137S–1143S. 10.3945/jn.114.194712 25833890 PMC4410494

[30] WHO. Serum ferritin concentrations for the assessment of iron status and iron deficiency in populations. Vitamin and Mineral Nutrition Information System. [Internet]. Vitamin and Mineral Nutrition Information System. Geneva; 2011. (WHO/NMH/NHD/MNM/11.2)

[31] Erhardt JG, Estes JE, Pfeiffer CM, Biesalski HK, Craft NE. Combined measurement of ferritin, soluble transferrin receptor, retinol binding protein, and C-reactive protein by an inexpensive, sensitive, and simple sandwich enzyme-linked immunosorbent assay technique. J Nutr. 2004;134: 3127–3132. 10.1093/jn/134.11.3127 15514286

[32] Haupt P. The SAARF Universal Living Standards Measure (SU-LSMTM): 12 years of continuous development [Internet]. 2016 [cited 25 Jul 2017]. Available: http://www.saarf.co.za/LSM/lsm-article.asp

[33] Stewart A, Marfell-Jones M, Olds T, De Ridder H. International standards for anthropometric assessment. Lower Hutt, New Zealand: International Society for the Advancement of Kinanthropmetry; 2011.

[34] Spong CY. Defining “Term” Pregnancy. JAMA. 2013; 10.1001/jama.2013.6235 23645117

[35] Butt K, Lim K, Bly S, Cargill Y, Davies G, Denis N, et al Determination of gestational age by ultrasound. J Obstet Gynaecol Canada. 2014;36: 171–181. 10.1016/S1701-2163(15)30664-2 24518917

[36] WHO Expert Committee on Diabetes Mellitus. Diabetes Mellitus. Geneva; 1985.

[37] Hsieh FY, Bloch DA, Larsen MD. A simple method of sample size calculation for linear and logistic regression. Stat Med. 1998;17: 1623–1634. Available: http://personal.health.usf.edu/ywu/logistic.pdf 9699234 10.1002/(sici)1097-0258(19980730)17:14<1623::aid-sim871>3.0.co;2-s

[38] Department of Justice and Constitutional Development. Getting married under customary law [Internet]. 2011 [cited 15 Jul 2019]. Available: http://www.justice.gov.za/services/getting-married-cusmar-law.html

[39] Institute of Medicine. Dietary Reference Intakes: the essential guide to nutrient requirements [Internet]. Washington, D.C: The National Academies Press; 2006 10.17226/11537

[40] Phatlhane D V, Zemlin AE, Matsha TE, Hoffmann M, Naidoo N, Ichihara K, et al The iron status of a healthy South African adult population. Clin Chim Acta. 2016;460: 240–245. 10.1016/j.cca.2016.06.019 27339094

[41] World Health Organization. Guideline: daily iron and folic acid supplementation in pregnant women [Internet]. Geneva; 2012. 10.1055/s-0028-1104741 23586119

[42] WHO. WHO recommendations on antenatal care for a positive pregnancy experience. Geneva, Switzerland; 2016. 28079998

[43] Fowkes FJI, Moore KA, Opi DH, Simpson JA, Langham F, Stanisic DI, et al Iron deficiency during pregnancy is associated with a reduced risk of adverse birth outcomes in a malaria-endemic area in a longitudinal cohort study. BMC Med. 2018;16: 156 10.1186/s12916-018-1146-z 30231938 PMC6149228

[44] Bah A, Pasricha S-R, Jallow MW, Sise EA, Wegmuller R, Armitage AE, et al Serum hepcidin concentrations decline during pregnancy and may identify iron deficiency: analysis of a longitudinal pregnancy cohort in The Gambia. J Nutr. 2017;147: 1131–1137. 10.3945/jn.116.245373 28424258 PMC5443464

[45] Vricella LK. Emerging understanding and measurement of plasma volume expansion in pregnancy. Am J Clin Nutr. 2017;106(Suppl): 1620S–1625S. 10.3945/ajcn. 117.155903 29070547 PMC5701717

[46] Fisher AL, Nemeth E. Iron homeostasis during pregnancy. Am J Clin Nutr. 2017;106: 1567S–1574S. 10.3945/ajcn.117.155812 29070542 PMC5701706

[47] Choi JW, Im MW, Pai SH. Serum Transferrin Receptor Concentrations during Normal Pregnancy. Clin Chem. 2000;46: 725–727. 10794761

[48] Fatima B, Demmouche A. Role of serum transferrin receptor in diagnosis of iron deficiency anemia: Report of 130 cases in West of Algeria. J Blood Disord Transfus. 2015;06: 4–9. 10.4172/2155-9864.1000305

[49] Beguin Y. Soluble transferrin receptor for the evaluation of erythropoiesis and iron status. Clin Chim Acta. 2003;329: 9–22. 10.1016/s0009-8981(03)00005-6 12589962

[50] Kelley-Hedgepeth A, Lloyd-Jones DM, Colvin A, Matthews KA, Johnston J, Sowers MR, et al Ethnic Differences in C-Reactive Protein Concentrations. Clin Chem. 2008;54: 1027–1037. 10.1373/clinchem.2007.098996 18403563

[51] Scheers N. Regulatory Effects of Cu, Zn, and Ca on Fe Absorption: The Intricate Play between Nutrient Transporters. Nutrients. 2013;5: 957–970. 10.3390/nu5030957 23519291 PMC3705329

[52] Petry N, Olofin I, Hurrell R, Boy E, Wirth J, Moursi M, et al The Proportion of Anemia Associated with Iron Deficiency in Low, Medium, and High Human Development Index Countries: A Systematic Analysis of National Surveys. Nutrients. 2016;8: 693 10.3390/nu8110693 27827838 PMC5133080

[53] Lao TT, Tam K-F, Chan LY. Third trimester iron status and pregnancy outcome in non-anaemic women; pregnancy unfavourably affected by maternal iron excess. Hum Reprod. 2000;15: 1843–1848. 10.1093/humrep/15.8.1843 10920115

[54] Shastri L, Mishra PE, Dwarkanath P, Thomas T, Duggan C, Bosch R, et al Association of oral iron supplementation with birth outcomes in non-anaemic South Indian pregnant women. Eur J Clin Nutr. Nature Publishing Group; 2015;69: 609–613. 10.1038/ejcn.2014.248 25406965

[55] Mwangi MN, Roth JM, Smit MR, Trijsburg L, Mwangi AM, Demir AY, et al Effect of daily antenatal iron supplementation on plasmodium infection in kenyan women: A randomized clinical trial. JAMA—J Am Med Assoc. 2015;314: 1009–1020. 10.1001/jama.2015.9496 26348751

[56] Imdad A, Bhutta ZA. Routine iron/folate supplementation during pregnancy: Effect on maternal anaemia and birth outcomes. Paediatr Perinat Epidemiol. 2012;26: 168–177. 10.1111/j.1365-3016.2012.01312.x 22742609

[57] Peña-Rosas J, De-Regil L, Carcia-Casal M, Dowswell T. Dialy oral iron supplementation during pregnancy. Cochrane Database Syst Rev. 2015; 10.1102/14651858.CD004736.pub5 PMC8918165 26198451

[58] Peña-Rosas JP, Viteri FE. Effects and safety of preventive oral iron or iron+folic acid supplementation for women during pregnancy. Cochrane Database Syst Rev. 2009; 223 10.1002/14651858.CD004736.pub3 19821332

[59] Hwang JY, Lee JY, Kim KN, Kim H, Ha EH, Park H, et al Maternal iron intake at mid-pregnancy is associated with reduced fetal growth: Results from Mothers and Children’s Environmental Health (MOCEH) study. Nutr J. 2013;12: 1–7. 10.1186/1475-2891-12-1 23547877 PMC3653712

[60] Brannon PM, Stover PJ, Taylor CL. Integrating themes, evidence gaps, and research needs identified by workshop on iron screening and supplementation in iron-replete pregnant women and young children. Am J Clin Nutr. 2017;106: 1703S–1712S. 10.3945/ajcn.117.156083 29070556 PMC5701718

[61] Etheredge AJ, Premji Z, Gunaratna NS, Abioye AI, Aboud S, Duggan C, et al Iron Supplementation in Iron-Replete and Nonanemic Pregnant Women in Tanzania. JAMA Pediatr. 2015;169: 947 10.1001/jamapediatrics.2015.1480 26280534 PMC4904713

[62] Friedrisch JR, Friedrisch BK. Prophylactic iron supplementation in pregnancy: a controversial issue. Biochem Insights. 2017;10: 117862641773773 10.1177/1178626417737738 29123406 PMC5661664

[63] Nairz M, Dichtl S, Schroll A, Haschka D, Tymoszuk P, Theurl I, et al Iron and innate antimicrobial immunity—Depriving the pathogen, defending the host. J Trace Elem Med Biol. Elsevier; 2018;48: 118–133. 10.1016/j.jtemb.2018.03.007 29773170

[64] Haider BA, Olofin I, Wang M, Spiegelman D, Ezzati M, Fawzi WW. Anaemia, prenatal iron use, and risk of adverse pregnancy outcomes: systematic review and meta-analysis. BMJ. 2013;346: f3443–f3443. 10.1136/bmj.f3443 23794316 PMC3689887

[65] Smith AM. Healthcare reform priorities for South Africa: four essays on the financing, delivery and user acceptability of healthcare by [Internet]. Stellenbosch University 2016 Available: http://ir.nrf.ac.za/bitstream/handle/10907/505/smith_healthcare_2016.pdf?sequence=1&isAllowed=y

