# Supplementary material for: Maternal iron-deficiency is associated with premature birth and higher birth weight despite routine antenatal iron supplementation in an urban South African setting: The NuPED prospective study
Source: PLoS One. 2019 Sep 3;14(9):e0221299. doi: 10.1371/journal.pone.0221299 (PMC6719862; doi:10.1371/journal.pone.0221299)
Supplement: S1 Table — IQR: interquartile range; CRP: C-reactive protein; AGP: α1-acid glycoprotein; LSM: Living Standards Measure; Hb: Haemoglobin; Fer: ferritin; sTfR: soluble transferrin receptor. Data are presented as n (%) for categorical variables and median (IQR) for continuous variables. ¥ Women who had a miscarriage or intrauterine foetal death (IUFD) also had missing birth weight data (n = 7), but were not considered in this table. † Mann-Whitney-U test for continuous variables, and Chi-square test for categorical variables. * n-values are equal to 250 for LSM, highest level of education, parity, HIV status, iron stores and iron deficiency erythropoiesis; 243 for anaemia; 239 for Country of birth; and 249 for all other variables. #: Traditional marriage, recognised under South African customary law, is entered between parties based on tradition which does not require the approval of an officiator for validation. It is also different from civil marriage in that a polygamous marriage is permissible. (DOCX) [file pone.0221299.s001.docx]

**S1 Table:** **Characteristics and iron status of pregnant women from the NuPED study at enrolment (<18 weeks of gestation) by birth weight data availability****^¥^**

|  | **Birth weight data obtained**  **84% (n=203)** | **Birth weight data missing**  **16% (n=40)** | ***p*†** | **Total sample  (n=250)*** |
| --- | --- | --- | --- | --- |
| ***Characteristics at enrolment*** |  |  |  | ***Median (IQR) or n (%)*** |
| **Age** (years) | 28 (24-32) | 27 (24-30) | 0.45 | 27 (24-32) |
| **Gestational age** (weeks) | 14 (12-16) | 14 (13-16) | 0.20 | 14 (12-16) |
| **BMI** (kg/m^2^) (n=242) | 26.3 (23.1-30.6) | 26.5 (22.2-33.7) | 0.98 | 26.3 (23.0-30.6) |
| Underweight (<18.5 kg/m^2^) | 7 (4) | 0 | 0.62 | 8 (3) |
| Normal weight (18.5-24.9 kg/m^2^) | 72 (36) | 15 (38) |  | 89 (36) |
| Overweight (25-29.9 kg/m^2^) | 67 (33) | 12 (30) |  | 81 (33) |
| Obese (≥30 kg/m^2^) | 56 (28) | 13 (33) |  | 71 (28) |
| **Ethnicity** (n=242) |  |  |  |  |
| Black African | 176 (87) | 36 (92) | 0.07 | 219 (88) |
| Mixed ancestry | 27 (13) | 2 (5) |  | 28 (11) |
| White | 1 (1) | 0 |  | 1 (<1) |
| Indian | 0 | 1 (3) |  | 1 (<1) |
| **Country of birth** (n=232) |  |  |  |  |
| South Africa | 140 (72) | 27 (71) | 0.15 | 172 (72) |
| Zimbabwe | 51 (86) | 8 (14) |  | 60 (25) |
| Lesotho | 2 (1) | 2 (5) |  | 4 (2) |
| Swaziland | 1 (1) | 1 (3) |  | 3 (1) |
| **Living Standards Measure (LSM)** (n=243) |  |  |  |  |
| Low (LSM 1-4) | 12 (6) | 4 (10) | 0.63 | 17 (7) |
| Medium (LSM 5-7) | 120 (59) | 23 (58) |  | 148 (59) |
| High (LSM 8-10) | 71 (35) | 13 (33) |  | 85 (34) |
| **Marital status** (n=242) |  |  |  |  |
| Unmarried/single | 84 (41) | 12 (31) | 0.29 | 100 (40) |
| Married | 51 (25) | 16 (41) |  | 68 (27) |
| Divorced/Separated | 2 (1) | 0 |  | 2 (1) |
| Living together | 47 (23) | 9 (23) |  | 57 (23) |
| Traditional marriage^#^ | 19 (8) | 2 (5) |  | 22 (9) |
| **Highest level of education** (n=242) |  |  |  |  |
| Primary school or less | 5 (3) | 4 (10) | 0.17 | 9 (4) |
| Grade 8 – 10 | 27 (13) | 8 (21) |  | 37 (15) |
| Grade 11 – 12 | 121 (60) | 20 (51) |  | 145 (58) |
| Post-school education | 50 (25) | 8 (18) |  | 58 (23) |
| **Parity** (n=243) |  |  |  |  |
| Nulliparous | 61 (30) | 10 (25) | 0.88 | 74 (30) |
| Primiparous | 70 (35) | 15 (38) |  | 88 (35) |
| Multiparous | 72 (35) | 15 (38) |  | 88 (35) |
| **HIV status** (n=243) |  |  |  |  |
| Positive | 49 (24) | 13 (33) | 0.27 | 64 (26) |
| Negative | 154 (76) | 27 (67) |  | 186 (74) |
| **Inflammatory status** (n=243) |  |  |  |  |
| Normal CRP | 83 (41) | 14 (35) | 0.49 |  |
| Elevated CRP (>5 mg/L) | 120 (59) | 26 (65) |  | 149 (60) |
| Normal AGP | 178 (88) | 38 (95) | 0.18 |  |
| Elevated AGP (>1 g/L) | 25 (12) | 2 (5) |  | 28 (11) |
| **Anaemia** (n=236) |  |  |  |  |
| Normal haemoglobin | 146 (75) | 22 (55) | **0.01** | 173 (71) |
| Anaemic (Hb <11g/dL) | 50 (25) | 18 (45) |  | 70 (29) |
| **Iron stores** (n=243) |  |  |  |  |
| Normal serum ferritin | 179 (88) | 28 (70) | **<0.01** | 213 (85) |
| Iron depleted (Fer <15 μg/L) | 24 (12) | 12 (30) |  | 37 (15) |
| **Iron deficiency erythropoiesis** (n=243) |  |  |  |  |
| Normal sTfR | 179 (85) | 33 (83) | 0.66 | 212 (85) |
| Increased sTfR (sTfR >8.3 mg/L) | 30 (15) | 7 (17) |  | 38 (15) |

IQR: interquartile range; CRP: C-reactive protein; AGP: α_1_-acid glycoprotein; LSM: Living Standards Measure; Hb: Haemoglobin; Fer: ferritin; sTfR: soluble transferrin receptor.

Data are presented as n (%) for categorical variables and median (IQR) for continuous variables.

¥ Women who had a miscarriage or intrauterine foetal death (IUFD) also had missing birth weight data (n=7), but were not considered in this table.

**†** Mann-Whitney-U test for continuous variables, and Chi-square test for categorical variables.

* n-values are equal to 250 for LSM, highest level of education, parity, HIV status, iron stores and iron deficiency erythropoiesis; 243 for anaemia; 239 for Country of birth; and 249 for all other variables.

#: Traditional marriage, recognised under South African customary law, is entered between parties based on tradition which does not require the approval of an officiator for validation. It is also different from civil marriage in that a polygamous marriage is permissible.
